# Circ_0078607 inhibits the progression of ovarian cancer via regulating the miR-32-5p/SIK1 network

**DOI:** 10.1186/s13048-021-00931-9

**Published:** 2022-01-04

**Authors:** Yangqiu Jin, Hui Wang

**Affiliations:** 1 Department of Reproductive Medicine, Liaocheng People’s Hospital, NO.67, Dongchang West Road, Liaocheng City, 252000 Shandong Province China; 2 Department of Obstetrics and Gynecology, Liaocheng Tird People’s Hospital, Liaocheng, Shandong China

**Keywords:** Ovarian cancer, circ_0078607, miR-32-5p, SIK1

## Abstract

**Background:**

Circular RNA (circRNA) has been shown to be involved in the regulation of human disease progression, including ovarian cancer (OC). Circ_0078607 was found to participate in OC progression. But its function and mechanism in OC deserve further exploration.

**Methods:**

The expression levels of circ_0078607, salt-inducible kinase 1 (SIK1) and microRNA (miR)-32-5p were examined by qRT-PCR. And the protein expression levels of SIK1, metastasis marker and apoptosis marker were determined using western blot analysis. EDU staining, colony formation assay, transwell assay and flow cytometry were used to detect the proliferation, migration, invasion and apoptosis of cells. Moreover, dual-luciferase reporter assay was employed to verify the interaction between miR-32-5p and circ_0078607 or SIK1. Xenograft models were constructed to perform in vivo experiments.

**Results:**

Circ_0078607 and SIK1 were downregulated in OC tissues and cells. Overexpressed circ_0078607 and SIK1 could inhibit OC cell proliferation, migration, invasion, and promote apoptosis. MiR-32-5p could be sponged by circ_0078607, and its overexpression could reverse the suppressive effect of circ_0078607 on OC progression. Furthermore, SIK1 was a target of miR-32-5p, and circ_0078607 could regulate SIK1 by sponging miR-32-5p. The inhibitory effect of circ_0078607 on OC progression also could be reversed by SIK1 silencing. In vivo experiments showed that circ_0078607 reduced OC tumorigenesis by regulating the miR-32-5p/SIK1 axis.

**Conclusion:**

Circ_0078607 could serve as a sponge of miR-32-5p to regulate SIK1 expression, thereby inhibiting OC progression.

**Supplementary Information:**

The online version contains supplementary material available at 10.1186/s13048-021-00931-9.

## Abstract (article highlights)

1. Circ_0078607 acts as a sponge of miR-32-5p.
2. MiR-32-5p targets SIK1.

**Supplementary Information:**

The online version contains supplementary material available at 10.1186/s13048-021-00931-9.

## Introduction

Ovarian cancer (OC) is a malignant tumor that occurs in the ovary and is the biggest disease that seriously threatens women’s health [9, 20]. Since most patients are in the advanced stage at the time of diagnosis, the treatment of OC is more difficult and the mortality of patients is high [12, 17]. Although the current treatment technology of OC continues to improve, the prognosis of most patients is still not ideal [4, 8]. Therefore, elucidating the molecular mechanisms affecting the occurrence of OC may provide a theoretical basis for finding effective therapeutic targets of OC.

As a special non-coding RNA, circular RNA (circRNA) has received a lot of attention in recent years. Studies have found that circRNA is widely and diversely present in a variety of biological cells and has the regulating effect on gene expression [1, 2]. Importantly, the researchers have found that the expression of circRNA is closely related to the progression of many human diseases, including OC [19, 22]. For example, circPLEKHM3 had been discovered to inhibit OC cell growth and metastasis [28]. On the contrary, circWHSC1 was upregulated in OC and was found to promote the proliferation, migration and invasion of OC [30]. Circ_0078607 is a newly discovered circRNA in recent years. Zhang et al. proposed that circ_0078607 was lowly expressed in OC and it might act as tumor suppressor to hinder OC progression [29]. However, there are still few studies on circ_0078607, and its role and mechanism in OC deserve further investigation.

Salt-inducible kinase 1 (SIK1) is a serine/threonine protein kinase, belongs to SIK family [24]. SIK1 is confirmed to have important function in regulating cell biological function and maintaining homeostasis [18, 26]. With the development of research, more and more studies have confirmed that SIK1 expression is also associated with the progression of many cancers, including hepatocellular carcinoma [15], pancreatic cancer [16] and colorectal cancer [7]. It had been reported that SIK1 knockdown could promote the proliferation of OC [3]. Therefore, SIK1 might play an anti-cancer role in OC.

CircRNA has been confirmed to act as a sponge of microRNA (miRNA), and then release miRNA’s inhibitory effect on mRNA to indirectly regulate mRNA expression [5, 13]. In our research, we found a significant negative correlation between circ_0078607 and SIK1. We speculated that there might be a miRNA that could interact with circ_0078607 and SIK1. Therefore, we conducted this research around the hypothesis of the circRNA/miRNA/mRNA axis.

## Materials and methods

### Tissue samples

A total of 43 patients diagnosed with OC were recruited from Liaocheng People’s Hospital. OC tumor tissues (*n* = 43) and adjacent normal tissues (*n* = 43) of all patients were collected after surgery. Informed consent was obtained from each patient. The study was approved by the Ethics Committee of Liaocheng People’s Hospital. The clinicopathologic features of OC patients were shown in Table 1.

**Table 1:** The correlation between circ_0078607 expression and clinicopathologic features of ovarian cancer patients

Characteristics	n = 43	Circ_0078607 expression		*P* value
		Low (*n* = 21)	High (*n* = 22)	
Age (years)				0.455
< 55	18	10	8	
≥55	25	11	14	
Histological type				0.083
I	14	10	4	
II-III	29	11	18	
FIGO stage				0.010*
I-II	20	14	6	
III	23	7	16	
Lymph node metastasis				0.021*
No	23	15	8	
Yes	20	6	14	
CA125				0.273
< 35	13	8	5	
≥35	30	13	17	

*FIGO* the International Federation of Gynecology and Obstetrics; *P < 0.05

### Cell culture and transfection

Human OC cell lines (HEY and ES-2) and normal epithelial ovarian cell line (IOSE80) were obtained from Biovector NTCC (Beijing, China). All cells were cultured in RPMI-1640 medium (Gibco, Grand Island, NY, USA) supplemented with 10% FBS (Gibco) and 1% double antibiotics (Gibco) at 37 °C in an incubator with 5% CO_2_. For cell transfection, HEY and ES-2 cells were seeded into 12-well plates. The circ_0078607 overexpression vector (oe-circ_0078607) and its control (NC), the pcDNA overexpression vector and small interference RNA (siRNA) of SIK1 (SIK1 and si-SIK1) or their controls (pc-DNA and si-NC), miR-32-5p mimic (miR-32-5p) and its control (miR-NC), as well as the lentivirus overexpression vector of circ_0078607 (lenti-circ_0078607) and its control (lenti-NC) were constructed by GenePharma (Shanghai, China) and were transfected into cells by Lipofectamine 3000 Reagent (Invitrogen, Carlsbad, CA, USA).

### qRT-PCR

The RNA were extracted by TRIzol reagent (Invitrogen). The cDNA was obtained using SuperScript IV First-Strand Synthesis System (Invitrogen). Basing on the specific primers, qRT-PCR was performed by SYBR Green (Solarbio, Beijing, China). Relative expression was examined using 2^−ΔΔCT^ method and normalized by GAPDH or U6. The sequences of primers were shown as follows: circ_0078607, F 5′-AGATCCTGAGACGCATTGCT-3′, R 5′-AGCCTAAGGTGAATGCTCCA-3′; SIK1, F 5′-CTCCGGGTGGGTTTTTACGAC-3′, R 5′-CTGCGTTTTGGTGACTCGATG-3′; miR-32-5p, F 5′-GCCGAGTATTGCACATTACTAA-3′, R 5′-GTGCAGGGTCCGAGGT-3′; GAPDH, F 5′-CTCTGCTCCTCCTGTTCGAC-3′, R 5′-CGACCAAATCCGTTGACTCC-3′; U6, F 5′-CTCGCTTCGGCAGCACATA-3′, R 5′-CGAATTTGCGTGTCATCCT-3′.

### Western blot (WB) analysis

RIPA buffer (Solarbio) was utilized for extracting total protein, and then the protein was quantified by BCA Kit (Sangon, Shanghai, China). Next, the proteins were separated by SDS-PAGE gel and electro-blotted to PVDF membrane (Beyotime, Shanghai, China). The membrane was incubated with the primary antibodies against SIK1 (51045–1-AP, 1:1000), E-cadherin (20874–1-AP, 1:5000), N-cadherin (22018–1-AP, 1:2000), matrix metalloproteinase 9 (MMP9, 10375–2-AP, 1:1000), Bcl2-associated x (Bax, 50599–2-Ig, 1:5000) or GAPDH (10494–1-AP, 1:20,000). After further incubating with Goat Anti-Rabbit IgG (SA00001–2, 1:10,000), the protein bands in the membranes were visualized using Ultrasensitive ECL Dection Kit (Proteintech, Rosemont, IL, USA). All antibodies were obtained from Proteintech.

### EDU staining

Click-iT® EDU Imaging Kit was obtained from Invitrogen. According to the kit instructions, the EDU positive cells were measured to evaluate cell proliferation.

### Colony formation assay

After transfection for 48 h, HEY and ES-2 cells were collected and then inoculated into 6-well plates. After culturing for 2 weeks, the cells were fixed with 4% paraformaldehyde and stained with crystal violet. Afterwards, the number of colonies was counted under a microscope (Leica, Wetzlar, Germany).

### Transwell assay

The transwell chambers (24-well, 8 μm) (Corning Inc., Corning, NY, USA) was used to measure cell migration and invasion. An additional Matrigel (Corning Inc.) was needed to pre-coat the upper chamber for detecting cell invasion. Briefly, HEY and ES-2 cells suspended in serum-free medium were placed in upper chambers. In addition, the complete medium was added into the lower chambers. 24 h later, the cells transferred to the lower chamber were fixed and stained, and its numbers were counted under a microscope (100 ×).

### Flow cytometry

Cell apoptosis was measured using the Annexin V-FITC/PI Apoptosis Detection Kit (BestBio, Shanghai, China). In brief, HEY and ES-2 cells were harvested after transfection for 48 h. The cells were then suspended with Binding Buffer, followed by staining with Annexin V-FITC and PI in the dark. Cell apoptosis rate was analyzed by flow cytometry and counted using the CellQuest software.

### Dual-luciferase reporter assay

The pGL3 luciferase reporter vectors for the wide-type (WT) and mutate-type (MUT) of circ_0078607 (WT/MUT-circ_0078607) or SIK1 3’UTR (WT/MUT-SIK1–3’UTR) were synthesized by GenePharma. The reporter vectors were co-transfected into HEY and ES-2 cells with miR-NC or miR-32-5p mimic. 48 h later, relative luciferase activity was detected using the Dual-Lucy Assay Kit (Solarbio).

### Xenograft models

The animal experiment was supported by the Animal Ethics Committee of Liaocheng People’s Hospital. Ten female BALB/c nude mice (Vital River, Beijing, China) were divided into 2 groups (*n* = 5 per group). HEY cells (5 × 10^6^ cells) transfected with lenti-NC or lenti-circ_0078607 were subcutaneously injected into the back of the nude mice. The tumor length and width were measured every 7 days to calculated tumor volume. After 28 days, the mice were euthanized and the tumor was collected. In addition, paraffin sections were prepared from tumor tissue to perform Ki-67 immunohistochemical (IHC) staining using Ki-67 Kit (Sangon).

### Statistical analysis

All experiment was performed in triplicate, and all independent experiments were set for 3 times to take the average value. All data were represented as mean ± standard deviation. Statistical analysis was performed using GraphPad Prism 7.0. Student’s *t*-test and one-way ANOVA followed by Tukey’s post-hoc test were used to analyze the differences. Pearson correlation analysis was used for analyzing the correlation among circ_0078607, miR-32-5p and SIK1. *P* < 0.05 was considered significant.

## Results

### Circ_0078607 and SIK1 were lowly expressed and positively correlated in OC tissues

**Fig. 1:**
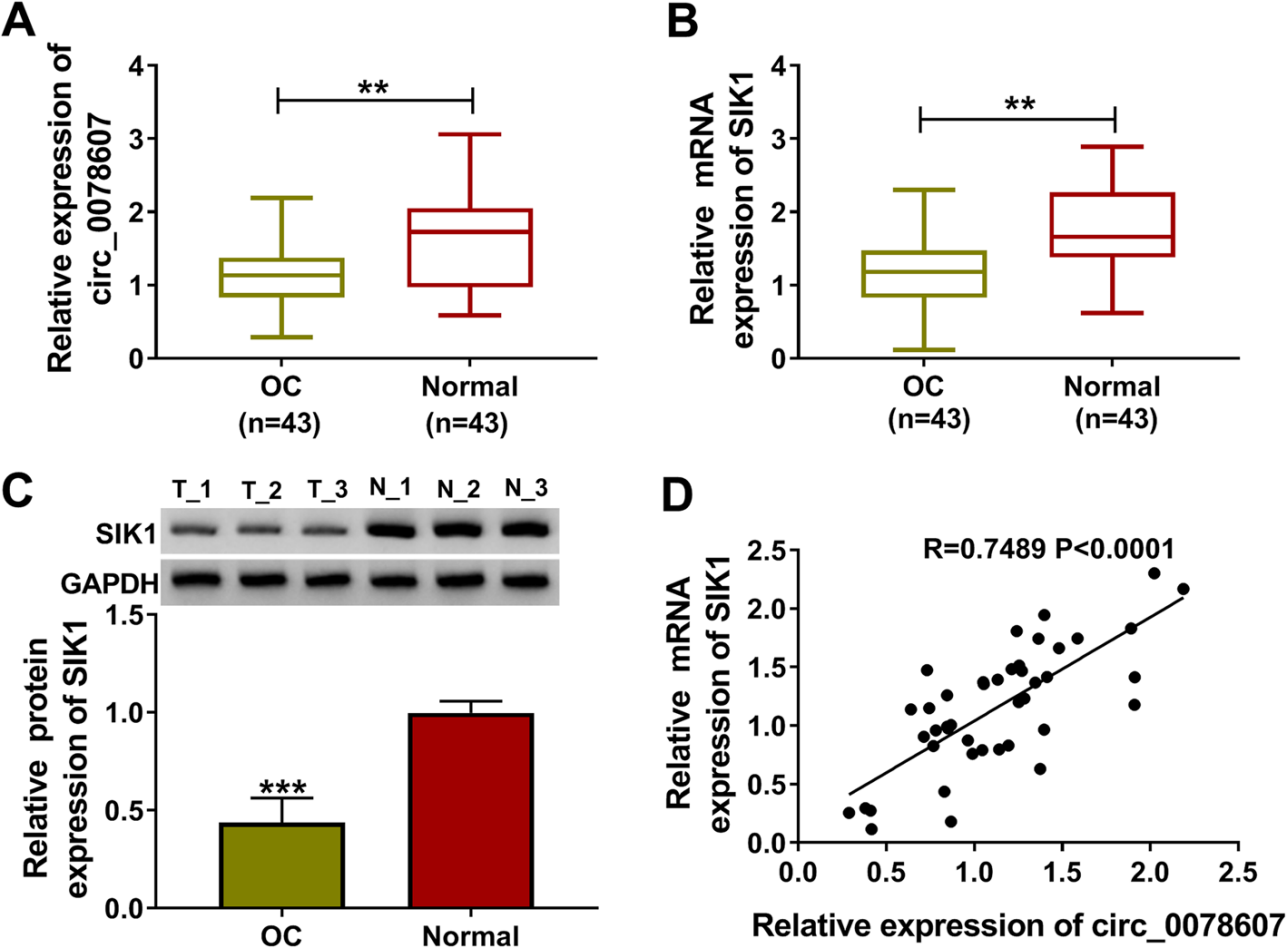
Circ_0078607 and SIK1 were lowly expressed and positively correlated in OC tissues. **A** The expression of circ_0078607 in OC tumor tissues (OC, *n* = 43) and adjacent normal tissues (Normal, n = 43) was detected by qRT-PCR. **B**-**C** The mRNA and protein expression levels of SIK1 in OC (*n* = 3) and Normal (n = 3) were measured by qRT-PCR and WB analysis. **D** Pearson correlation analysis was used to assess the correlation between circ_0078607 and SIK1 in OC tumor tissues (n = 43). **A**-**C**, Student’s *t*-test. **P < 0.01, ***P < 0.001

In OC tumor tissues, we discovered that circ_0078607 was downregulated compared with that in adjacent normal tissues (Fig. 1A). Then, we divided the 43 OC patients into low and high circ_0078607 expression groups according to the median expression level of circ_0078607. Through analyzing the correlation between circ_0078607 expression and clinicopathologic features of OC patients, we discovered that circ_0078607 expression was correlated with the FIGO stage and lymph node metastasis of OC patients (Table 1). Besides, SIK1 expression also was lower in OC tumor tissues than that in adjacent normal tissues at the mRNA level and protein level (Fig. 1B-C). Surprisingly, the correlation analysis results showed that there was a significant positive correlation between the expression of circ_0078607 and SIK1 in OC tumor tissues (Fig. 1D). These results suggested that circ_0078607 and SIK1 might play the important roles in the progression of OC.

### The expression of circ_0078607 and SIK1 was decreased in OC cells

**Fig. 2:**
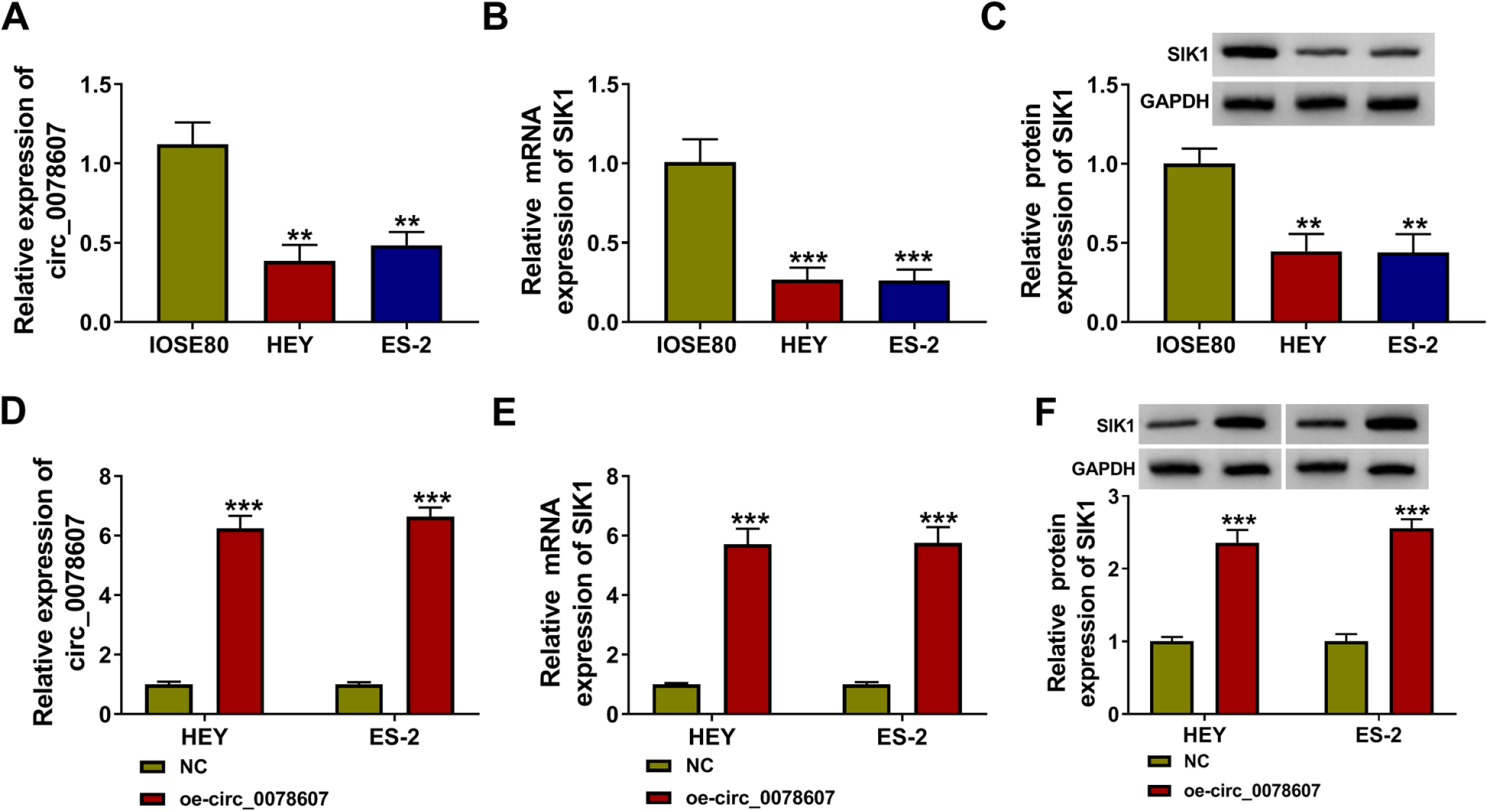
The expression of circ_0078607 and SIK1 was decreased in OC cells. **A** QRT-PCR was used to measure the expression of circ_0078607 in IOSE80 cells and both OC cells (HEY and ES-2). **B**-**C** QRT-PCR and WB analysis were performed to detect the mRNA and protein expression of SIK1 in IOSE80 cells and both OC cells (HEY and ES-2). **D** After transfecting with NC and oe-circ_0078607 into HEY and ES-2 cells, the expression of circ_0078607 was tested by qRT-PCR. **E**-**F** The mRNA and protein expression of SIK1 in HEY and ES-2 cells transfected with NC and oe-circ_0078607 were determined using qRT-PCR and WB analysis. **P < 0.01, ***P < 0.001

At the same time, we measured circ_0078607 and SIK1 expression on OC cells. The results showed that compared to the IOSE80 cells, circ_0078607 expression and SIK1 mRNA and protein expression were markedly lowly expressed in OC cells (HEY and ES-2) (Fig. 2A-C). To confirm the regulation of circ_0078607 on SIK1, we constructed the circ_0078607 overexpression vector. After transfecting with oe-circ_0078607 into HEY and ES-2 cells, circ_0078607 expression was significantly enhanced (Fig. 2D). The detection results of SIK1 mRNA and protein expression suggested that circ_0078607 overexpression could remarkably promote SIK1 expression in HEY and ES-2 cells (Fig. 2E-F). Our data showed that circ_0078607 positively regulated SIK1 in OC cells.

### Overexpressed circ_0078607 suppressed proliferation, migration, invasion and promoted apoptosis in OC cells

**Fig. 3:**
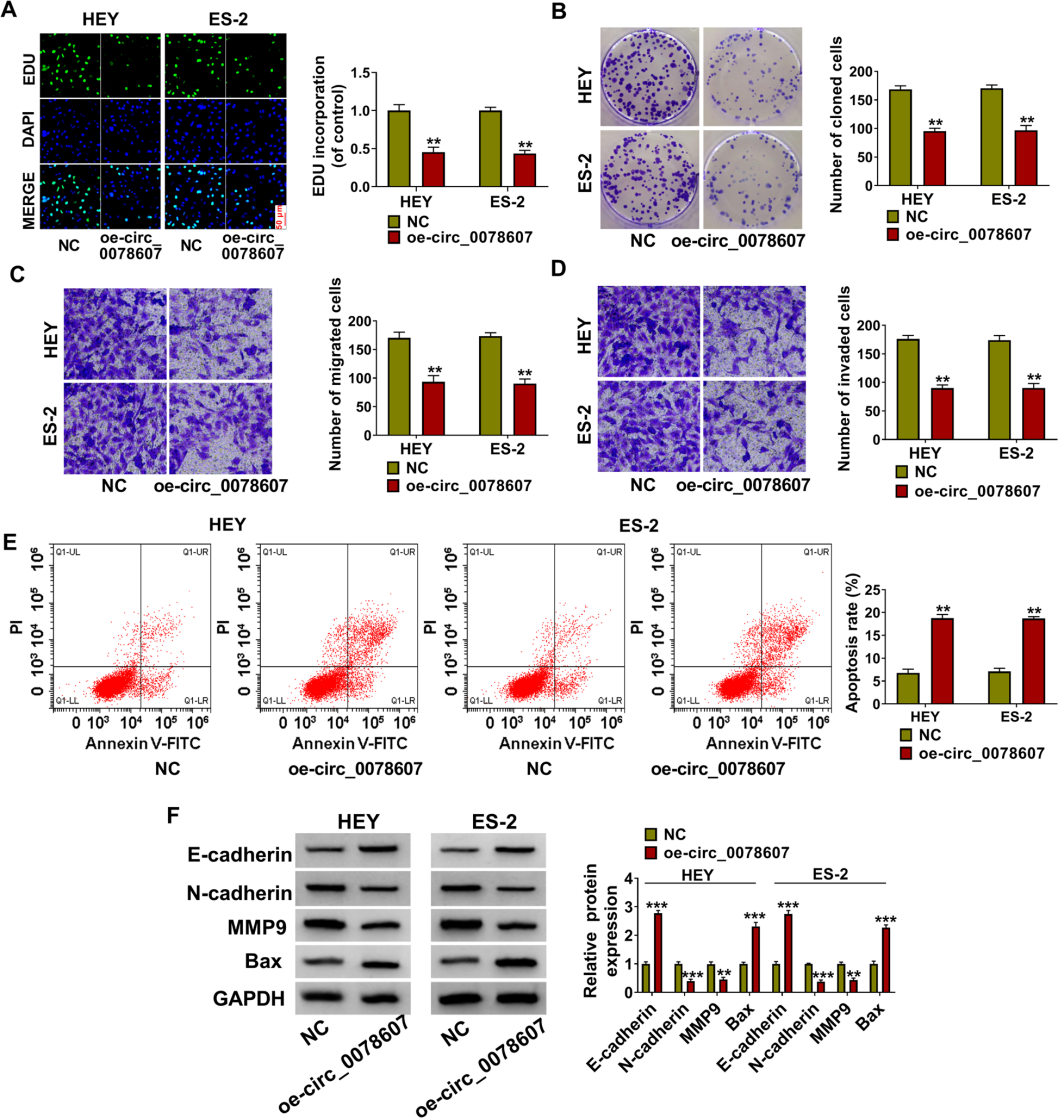
Overexpressed circ_0078607 suppressed OC cell progression. HEY and ES-2 cells were transfected with NC or oe-circ_0078607. EDU staining (**A**), colony formation assay (**B**), transwell assay (**C**-**D**) and flow cytometry (**E**) were used to measure the EDU positive cells, the number of colonies, the numbers of migrated and invaded cells, and the apoptosis rate of cells, respectively. **F** The protein expression levels of E-cadherin, N-cadherin, MMP9 and Bax were examined using WB analysis. **P < 0.01, ***P < 0.001

Then, we assessed the biological functions of HEY and ES-2 cells after overexpressing circ_0078607. EDU staining and colony formation assay results suggested that circ_0078607 overexpression could inhibit the EDU positive cells and reduce the number of cloned HEY and ES-2 cells (Fig. 3A-B), showing that the proliferation of OC cells could be repressed by circ_0078607 overexpression. Besides, the numbers of migrated and invaded HEY and ES-2 cells also were obviously inhibited by circ_0078607 overexpression (Fig. 3C-D). The results of flow cytometry indicated that overexpressed circ_0078607 could enhance the apoptosis rate of HEY and ES-2 cells (Fig. 3E). In addition, the protein levels of N-cadherin and MMP9 were markedly decreased, while the protein levels of E-cadherin and Bax were notably increased in HEY and ES-2 cells overexpressed circ_0078607 (Fig. 3F). These data indicated that circ_0078607 might play a negative role in OC progression.

### SIK1 overexpression inhibited OC progression

**Fig. 4:**
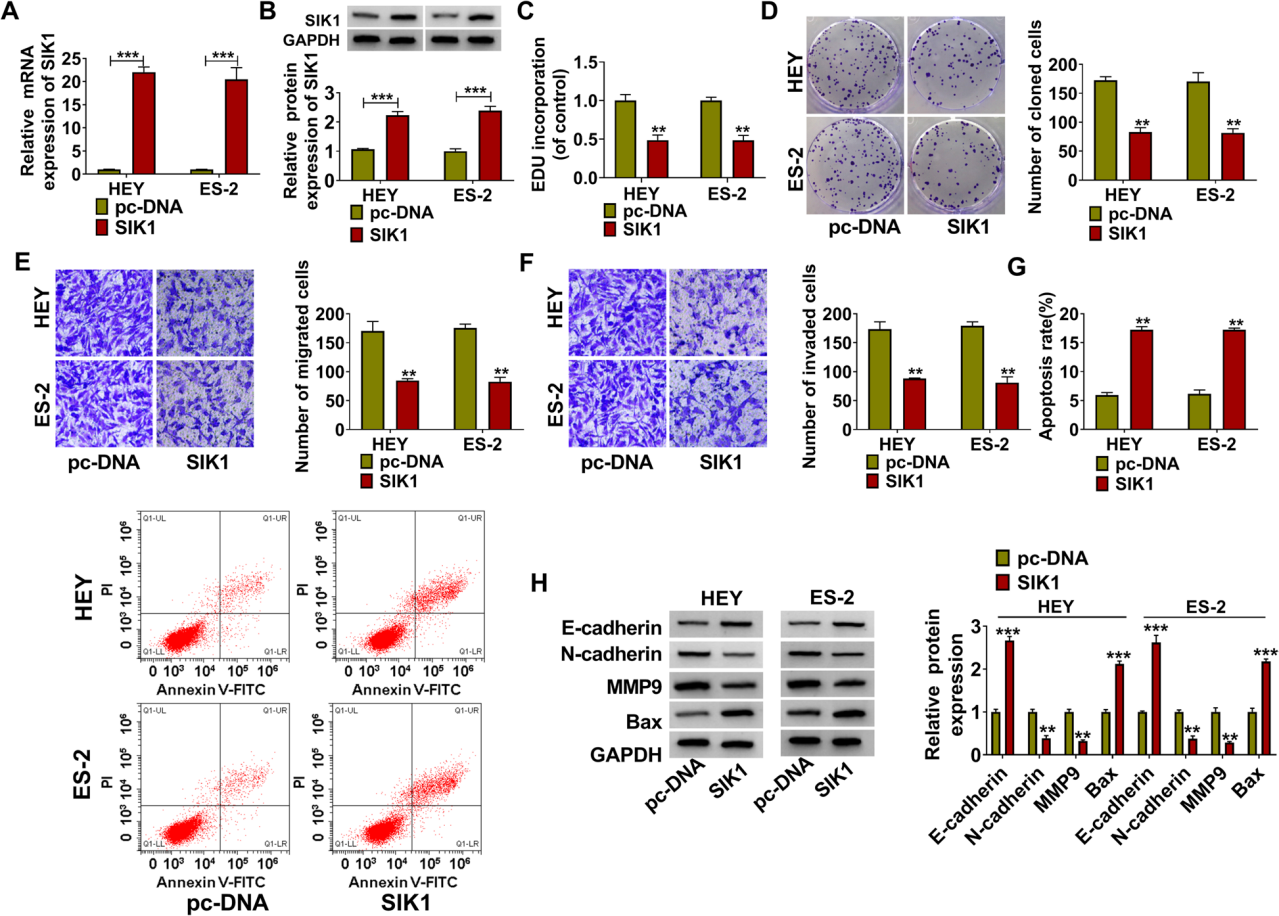
SIK1 overexpression inhibited OC progression. HEY and ES-2 cells were transfected with pc-DNA or pc-DNA SIK1 overexpression vector (SIK1). **A**-**B** The mRNA and protein expression levels of SIK1 were examined using qRT-PCR and WB analysis. The EDU positive cells, the number of colonies, the numbers of migrated and invaded cells, and the apoptosis rate of cells were determined using EDU staining (**C**), colony formation assay (**D**), transwell assay (**E**-**F**) and flow cytometry (**G**). **H** WB analysis was used to detect the protein expression levels of E-cadherin, N-cadherin, MMP9 and Bax. **P < 0.01, ***P < 0.001

To explore the role of SIK1 in OC progression, we also built the pc-DNA overexpression vector of SIK1. By detecting SIK1 mRNA and protein expression after transfection, we confirmed that pc-DNA SIK1 overexpression vector indeed increased SIK1 expression (Fig. 4A-B), indicating the successful transfection. Subsequently, we evaluated the effect of SIK1 overexpression on OC cell progression. Functional experiments showed that overexpressed SIK1 could significantly inhibit EDU positive cells, the number of colonies and the numbers of migrated and invaded cells in HEY and ES-2 cells (Fig. 4C-F). Also, SIK1 overexpression promoted the apoptosis rate, inhibited N-cadherin and MMP9 protein levels, while accelerated E-cadherin and Bax protein levels in HEY and ES-2 cells (Fig. 4G-H). Therefore, our data revealed that SIK1 might hinder OC progression, which was similar to the role of circ_0078607 in OC.

### Circ_0078607 could act as a sponge of miR-32-5p

**Fig. 5:**
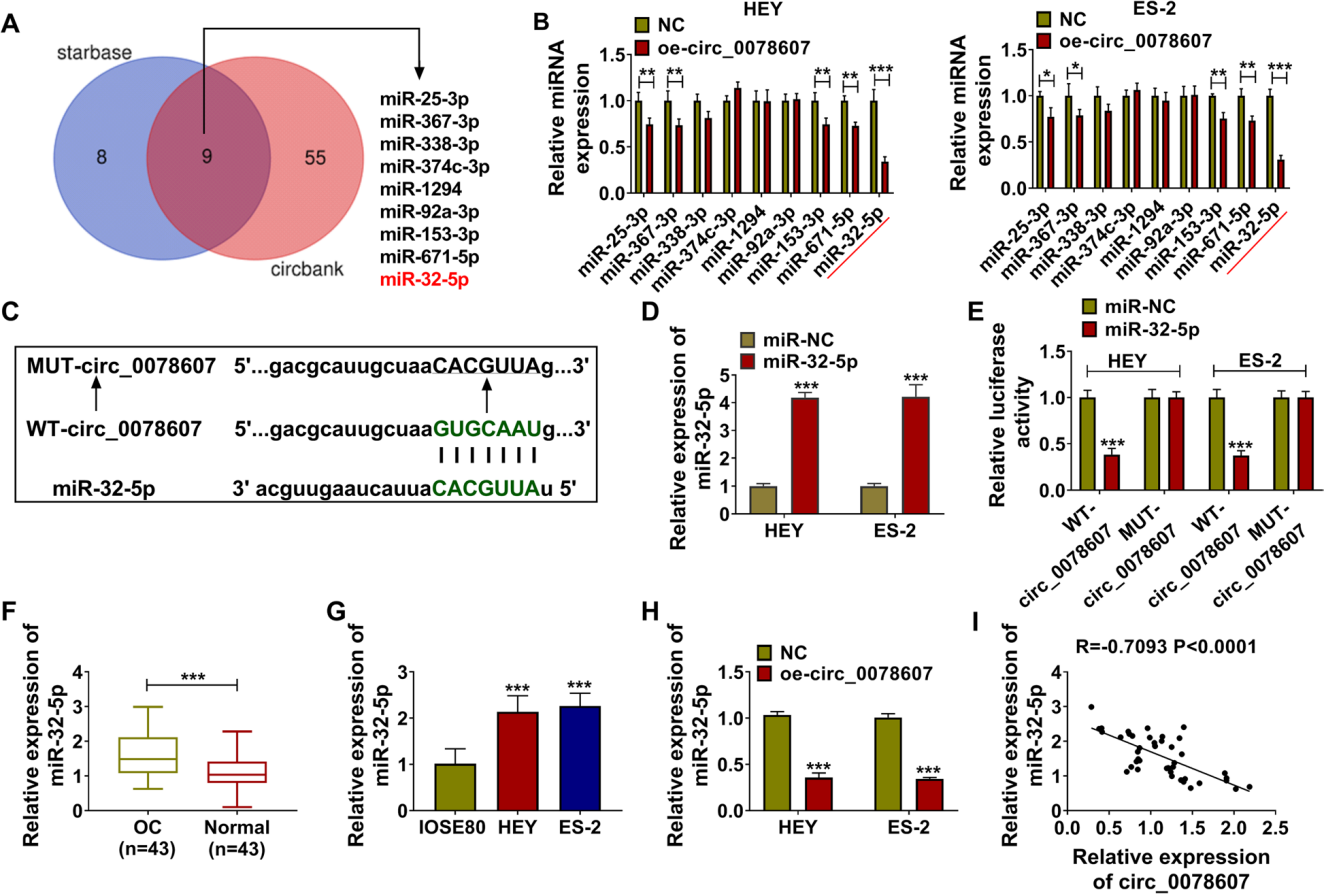
Circ_0078607 could act as a sponge of miR-32-5p. **A** Venn diagram shows candidate miRNAs for circ_0078607. **B** The expression of candidate miRNAs was detected by qRT-PCR in HEY and ES-2 cells transfected with NC or oe-circ_0078607. **C** The binding sites between circ_0078607 and miR-32-5p were shown. **D** The transfection efficiency of miR-32-5p mimic in HEY and ES-2 cells was assessed by detecting miR-32-5p expression using qRT-PCR. **E** Dual-luciferase reporter assay was performed to verify the interaction between circ_0078607 and miR-32-5p. **F** QRT-PCR was used to measure the expression of miR-32-5p in OC tumor tissues (OC, *n* = 43) and adjacent normal tissues (Normal, n = 43). **G** MiR-32-5p expression in IOSE80 cells and both OC cells (HEY and ES-2) was tested by qRT-PCR. **H** The expression of miR-32-5p in HEY and ES-2 cells transfected with NC or oe-circ_0078607 was detected by qRT-PCR. **I** The correlation between circ_0078607 and miR-32-5p in OC tumor tissues (n = 43) was analyzed using Pearson correlation analysis. ***P < 0.001

To explore the targeted miRNA of circ_0078607, we used the starbase and circbank software to perform bioinformatic prediction, and a total of 9 candidate miRNAs were screened out (Fig. 5A). After overexpression of circ_0078607, we found that miR-32-5p expression was the most obvious inhibited (Fig. 5B), so miR-32-5p was selected as the target miRNA of circ_0078607 for this study. According to the binding sites, the WT/MUT-circ_0078607 vectors were generated (Fig. 5C). To carry out follow-up experiments, we constructed miR-32-5p mimic and confirmed that it could indeed promote miR-32-5p expression in HEY and ES-2 cells (Fig. 5D). Besides, dual-luciferase reporter assay was used to verify the interaction between miR-32-5p and circ_0078607, and the results showed that miR-32-5p overexpression could obviously reduce the luciferase activity of WT-circ_0078607 vector without affecting that of the MUT-circ_0078607 vector (Fig. 5E). In OC tumor tissues and cells, we discovered that miR-32-5p expression was remarkably increased compared with that in the adjacent normal tissues and IOSE80 cells, respectively (Fig. 5F-G). And miR-32-5p expression could be obviously inhibited by circ_0078607 overexpression in HEY and ES-2 cells (Fig. 5H). Through the Pearson correlation analysis, we found that there had a negative correlation between miR-32-5p and circ_0078607 in OC tumor tissues (Fig. 5I). These results illuminated that miR-32-5p was a targeted miRNA of circ_0078607.

### MiR-32-5p reversed the regulation of circ_0078607 on OC proliferation, metastasis and apoptosis

**Fig. 6:**
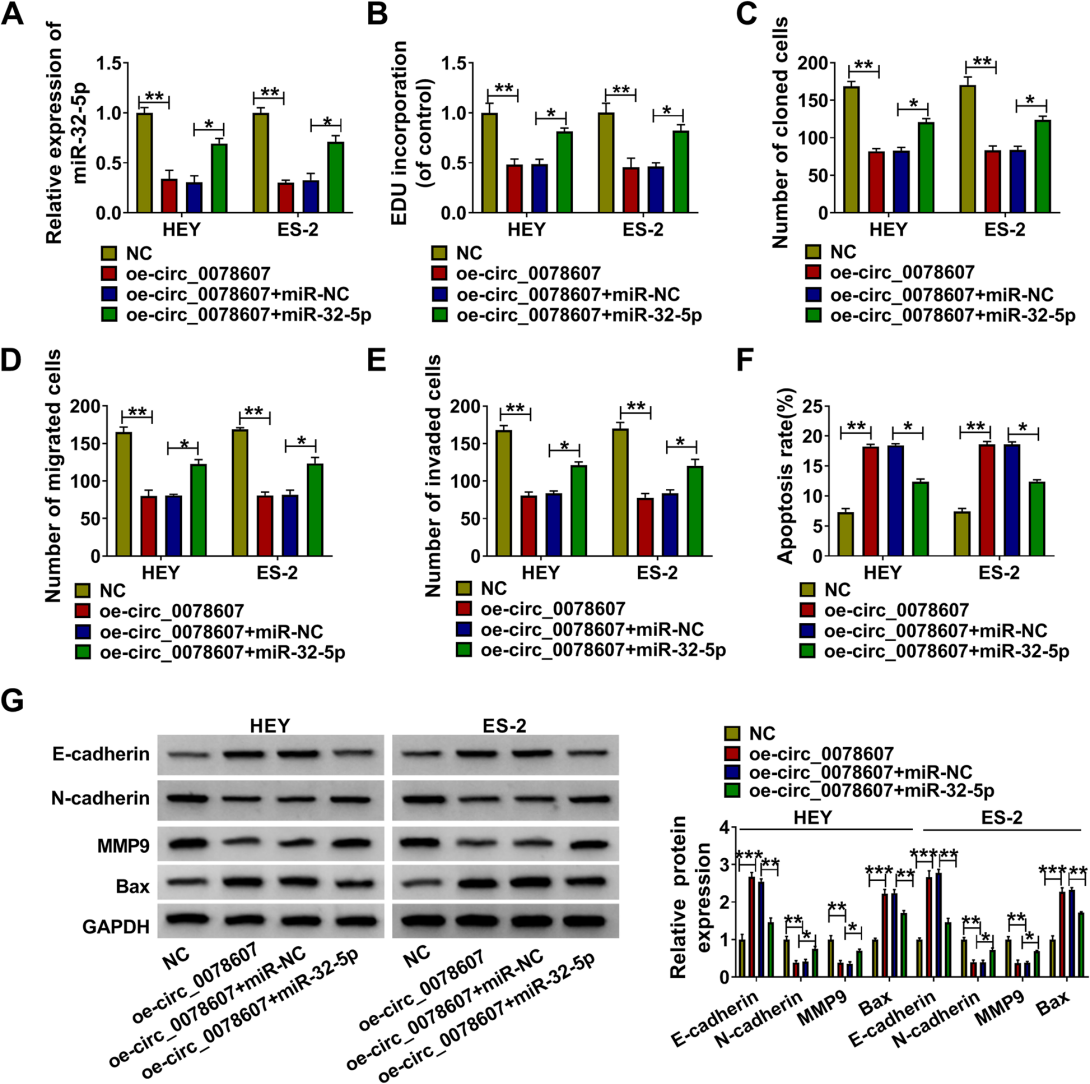
MiR-32-5p reversed the regulation of circ_0078607 on OC progression. HEY and ES-2 cells were transfected with NC, oe-circ_0078607, oe-circ_0078607 + miR-NC or oe-circ_0078607 + miR-32-5p. **A** The expression of miR-32-5p was detected using qRT-PCR. EDU staining (**B**), colony formation assay (**C**), transwell assay (**D**-**E**) and flow cytometry (**F**) were performed to measure the EDU positive cells, the number of colonies, the numbers of migrated and invaded cells, and the apoptosis rate of cells, respectively. **G** WB analysis was employed to determine the protein expression of E-cadherin, N-cadherin, MMP9 and Bax. *P < 0.05, **P < 0.01, ***P < 0.001

To further confirm that circ_0078607 regulated OC progression via sponging miR-32-5p, oe-circ_0078607 and miR-32-5p mimic were co-transfected into HEY and ES-2 cells. Through detecting miR-32-5p, we confirmed that the addition of miR-32-5p mimic could promote miR-32-5p expression inhibited by circ_0078607 overexpression (Fig. 6A). EDU staining, colony formation assay and transwell assay results showed that the suppressive effect of circ_0078607 overexpression on EDU positive cells, the number of colonies and the numbers of migrated and invaded cells could be reversed by miR-32-5p overexpression (Fig. 6B-E and Supplementary Fig. 1A-C). Furthermore, the apoptosis rate of HEY and ES-2 cells promoted by circ_0078607 also could be abolished by the addition of miR-32-5p mimic (Fig. 6F and Supplementary Fig. 1D). The decreasing effect of circ_0078607 on N-cadherin and MMP9 protein levels, as well as the increasing effect on E-cadherin and Bax protein levels also were recovered by overexpressing miR-32-5p (Fig. 6G). All data revealed that miR-32-5p was involved in the regulation of circ_0078607 on OC progression.

### SIK1 was a target of miR-32-5p

**Fig. 7:**
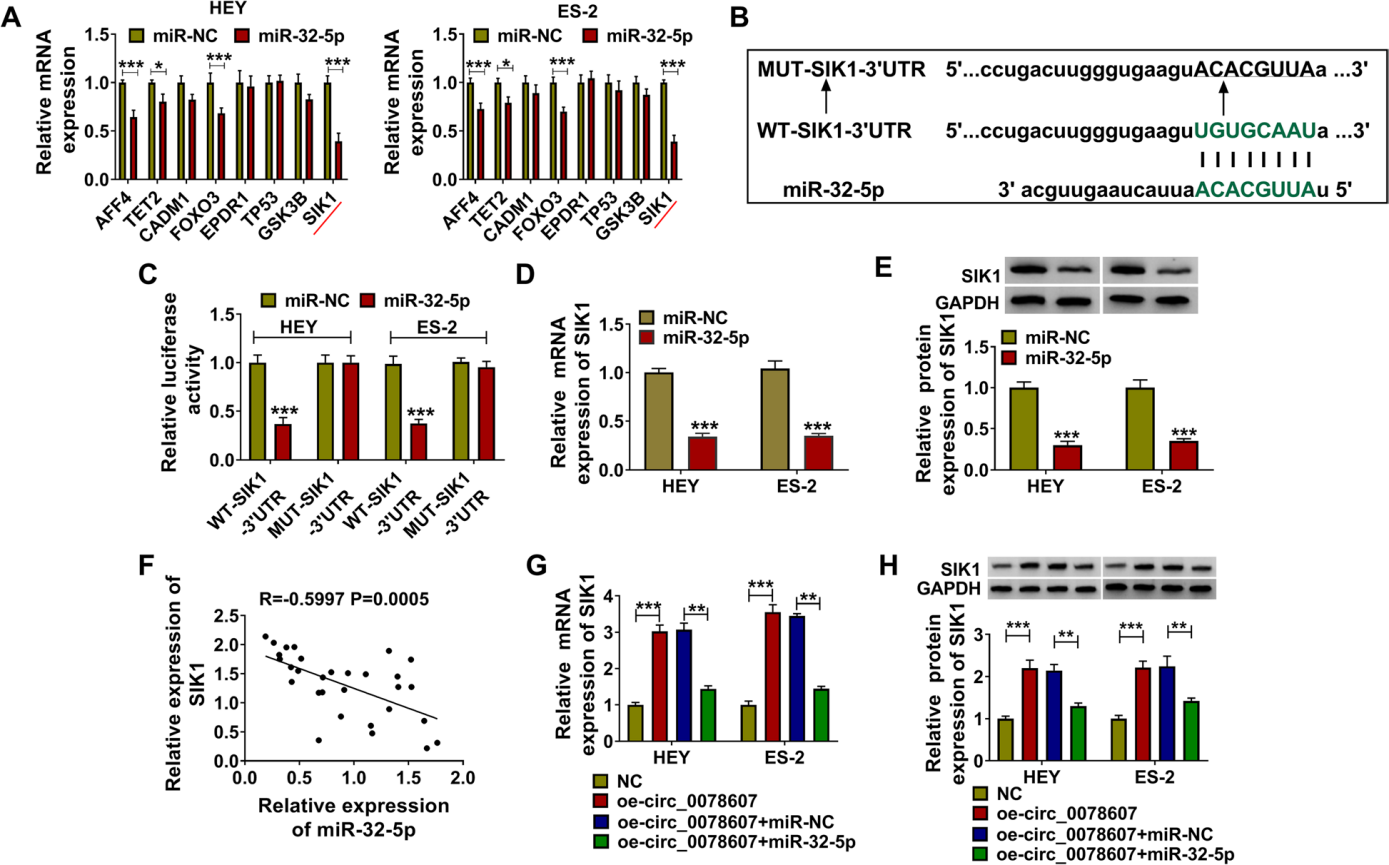
SIK1 was a target of miR-32-5p. **A** The expression of candidate genes was detected by qRT-PCR in HEY and ES-2 cells transfected with miR-NC or miR-32-5p mimic. **B** The binding sites between SIK1 3’UTR and miR-32-5p were exhibited. **C** Dual-luciferase reporter assay was used to confirm the interaction between SIK1 and miR-32-5p. **D**-**E** QRT-PCR and WB analysis were used to measure the mRNA and protein expression of SIK1 in HEY and ES-2 cells transfected with miR-NC or miR-32-5p. **F** The correlation between SIK1 and miR-32-5p in OC tumor tissues (n = 43) was assessed by Pearson correlation analysis. **G**-**H** HEY and ES-2 cells were transfected with NC, oe-circ_0078607, oe-circ_0078607 + miR-NC or oe-circ_0078607 + miR-32-5p. The mRNA and protein expression levels of SIK1 were examined using qRT-PCR and WB analysis. **P < 0.01, ***P < 0.001

Using several online databases, we selected 8 genes that have been reported to play tumor suppressor function in OC as candidate genes. QRT-PCR was used to detect the influence of miR-32-5p overexpression on the mRNA expression of these genes, and it was found that miR-32-5p overexpression had the most significant inhibition on SIK1 expression (Fig. 7A). Therefore, SIK1 was used as the target of miR-32-5p in this study. The sequences of WT/MUT-SIK1–3’UTR vector were shown in Fig. 7B. The results of dual-luciferase reporter assay suggested that the luciferase activity of WT-SIK1–3’UTR vector could be inhibited by miR-32-5p mimic, while that of the MUT-SIK1–3’UTR vector was not affected by miR-32-5p mimic (Fig. 7C). Moreover, we found that the mRNA and protein expression levels of SIK1 could be reduced by miR-32-5p overexpression (Fig. 7D-E). In OC tumor tissues, SIK1 mRNA expression was discovered to be negatively correlated with miR-32-5p expression (Fig. 7F). Furthermore, we also uncovered that the promoting effect of circ_0078607 overexpression on the mRNA and protein expression levels of SIK1 could be reversed by miR-32-5p overexpression (Fig. 7G-H). Therefore, we confirmed that circ_0078607 sponged miR-32-5p to regulate SIK1.

### The negative regulation of circ_0078607 on OC progression could be reversed by SIK1 silencing

**Fig. 8:**
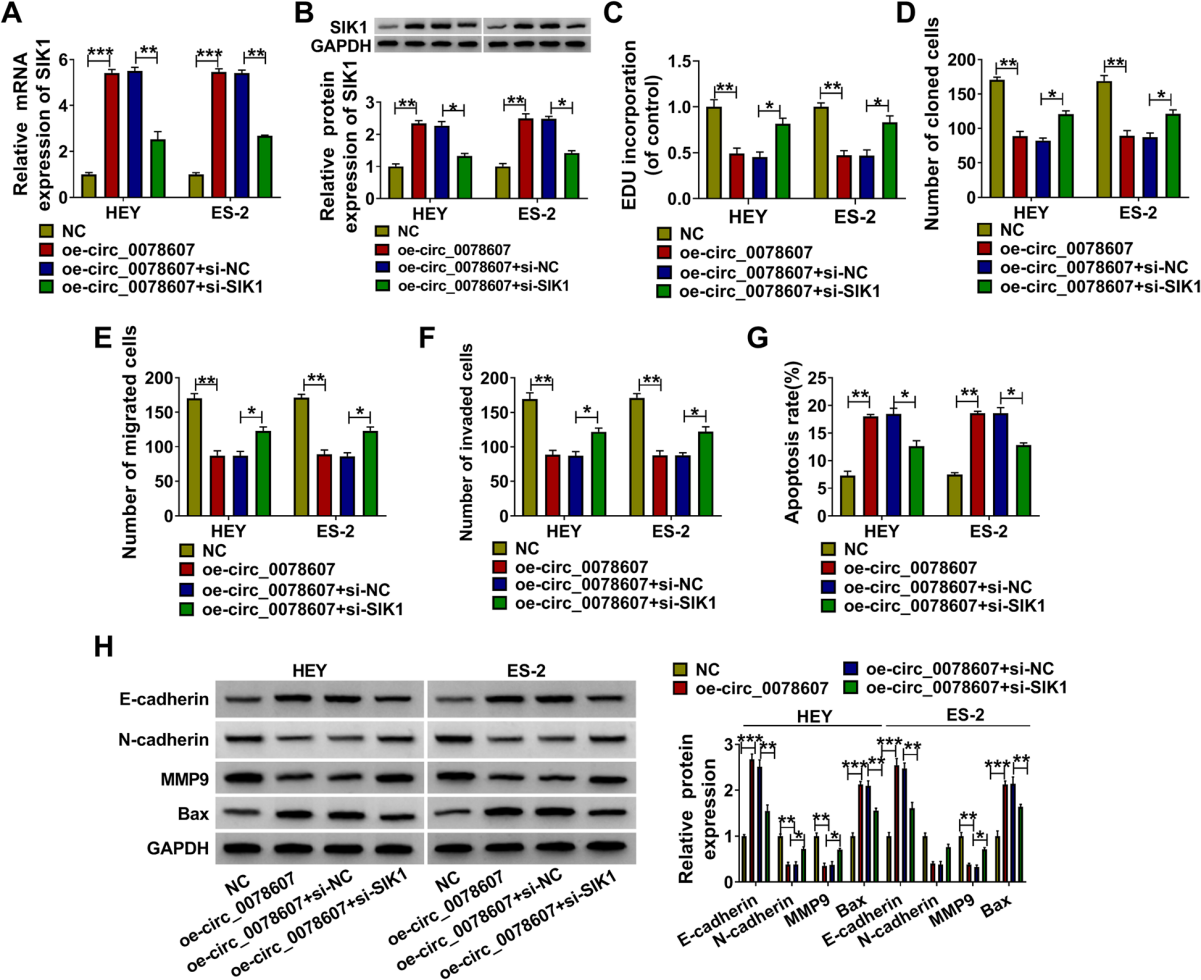
The negative regulation of circ_0078607 on OC progression could be reversed by SIK1 silencing. HEY and ES-2 cells were transfected with NC, oe-circ_0078607, oe-circ_0078607 + si-NC or oe-circ_0078607 + si-SIK1. **A**-**B** QRT-PCR and WB analysis were used to test the mRNA and protein expression of SIK1. The EDU positive cells, the number of colonies, the numbers of migrated and invaded cells, and the apoptosis rate of cells were determined using EDU staining (**C**), colony formation assay (**D**), transwell assay (**E**-**F**) and flow cytometry (**G**). **H** The protein expression levels of E-cadherin, N-cadherin, MMP9 and Bax were evaluated by WB analysis. *P < 0.05, **P < 0.01, ***P < 0.001

To further confirm that the regulation of circ_0078607 on OC progression was achieved by mediating SIK1 expression, oe-circ_0078607 and si-SIK1 were co-transfected into HEY and ES-2 cells. Through measuring the mRNA and protein expression levels of SIK1, we found that the enhancing effect of circ_0078607 on SIK1 expression could be inhibited by si-SIK1 (Fig. 8A-B). Further experiments revealed that knockdown of SIK1 could abolish the inhibitory effects of circ_0078607 overexpression on the EDU positive cells, the number of colonies and the numbers of migrated and invaded cells (Fig. 8C-F and Supplementary Fig. 2A-C). Besides, silenced SIK1 also reversed the increasing effect of circ_0078607 overexpression on the apoptosis rate of HEY and ES-2 cells (Fig. 8G and Supplementary Fig. 2D). The reducing effect of circ_0078607 on N-cadherin and MMP9 protein levels, as well as the promoting effect on E-cadherin and Bax protein levels also could be recovered by SIK1 knockdown in HEY and ES-2 cells (Fig. 8H). Our data showed that circ_0078607 regulated OC progression by regulating SIK1.

### Circ_0078607 overexpression inhibited OC tumorigenesis

**Fig. 9:**
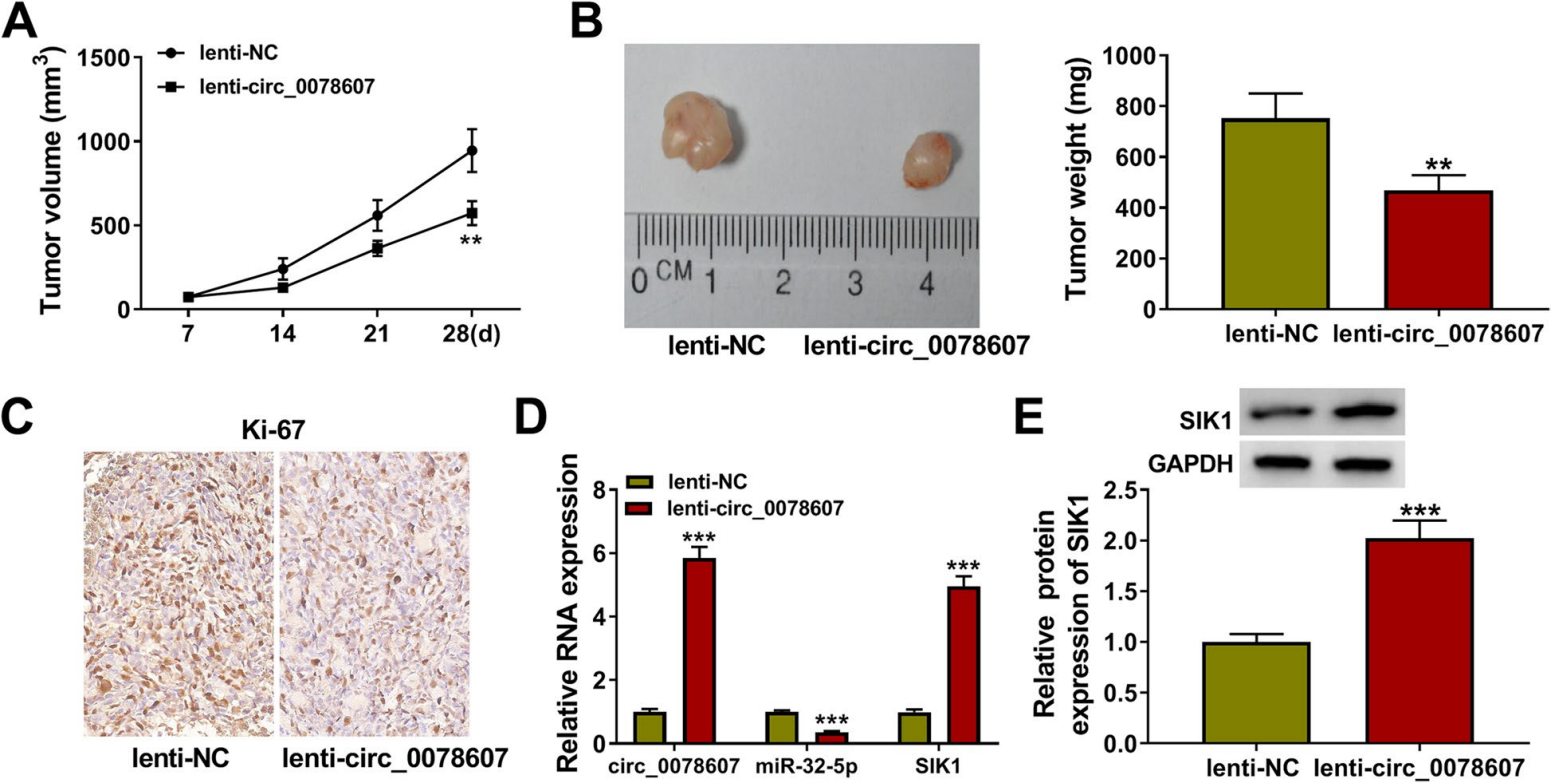
Circ_0078607 overexpression inhibited OC tumorigenesis in vivo*.* HEY cells transfected with lenti-NC or lenti-circ_0078607 were injected into nude mice (*n* = 5). **A** Tumor volume was measured every 7 days until 28 days. **B** After 28 days, the tumors were removed and weighted. **C** IHC staining was used to evaluate the Ki-67 positive cells in the tumor tissues. **D** The expression of circ_0078607, miR-32-5p and SIK1 in the tumors was detected using qRT-PCR. **E** The protein expression of SIK1 in the tumors was assessed using WB analysis. **P < 0.01, ***P < 0.001

For further investigating the role of circ_0078607 in OC, we assessed the effect of circ_0078607 overexpression on OC tumorigenesis in vivo. After 28 days of subcutaneous xenograft tumors construction, we found that tumor volume and weight in the circ_0078607 overexpression group were significantly lower than those in the control group (Fig. 9A-B). IHC staining results showed that the Ki-67 positive cells were markedly reduced in the tumor tissues of the lenti-circ_0078607 group (Fig. 9C). By detecting circ_0078607 expression in the tumors, we confirmed that circ_0078607 was indeed overexpressed in the circ_0078607 overexpression group (Fig. 9D). At the same time, we also found that miR-32-5p expression was obviously reduced, while SIK1 mRNA and protein expression levels were markedly enhanced in the circ_0078607 overexpression group (Fig. 9D-E). All data indicated that circ_0078607 suppressed OC tumorigenesis by regulating the miR-32-5p/SIK1 axis.

## Discussion

In many studies, circRNA has been confirmed to be associated with cancer malignant progression, and different circRNA has different roles in different cancers. Li et al. showed that circTGFBR2 could hinder nasopharyngeal carcinoma proliferation and migration via sponging miR-107 [10]. CircCUL2 was downregulated in gastric cancer and it could repress cancer malignant transformation and promote cisplatin sensitivity by sponging miR-142-3p and regulating ROCK2 [14]. In addition, circ_100146 had been discovered to be upregulated in bladder cancer, which could facilitate cell proliferation and metastasis via the miR-149-5p/RNF2 axis [21]. However, there are few studies on circ_0078607. Our study showed that circ_0078607 was underexpressed in OC tissues and cells, and it had an anti-proliferation, anti-metastasis, and pro-apoptosis function in OC. Animal experiments also indicated that circ_0078607 overexpression also could reduce OC tumorigenesis in vivo. Our data proposed that circ_0078607 play a negative role in OC progression, which was consistent with the reported results of Zhang et al. [29].

In previous studies, SIK1 has been found to have highly effective in preventing tumor formation and cancer progression [6, 25]. In our study, we discovered a significant positive correlation between circ_0078607 and SIK1 with low expression in OC tumor tissues. SIK1 had been proved to be a tumor suppressor to regulate OC progression, which was similar to the effect of circ_0078607 on OC progression and consistent with the previous research results [3]. The analysis result of the functional test showed that SIK1 had an inhibitory effect on OC cell proliferation and metastasis, and could promote cell apoptosis. Considering that circRNA can indirectly regulate mRNA expression by sponging miRNA, we hypothesized that there was an interacted miRNA, which could be sponged by circ_0078607 and could target SIK1. After bioinformatics analysis and screening, we locked miR-32-5p.

MiR-32-5p has different functions in different cancers. Liang et al. showed that miR-32-5p had high expression in colorectal cancer, which could promote cancer cell metastasis and radioresistance [11]. In contrast, miR-32-5p was discovered to restrain breast cancer proliferation and increase apoptosis [23]. In OC, miR-32-5p was found to enhance OC proliferation and metastasis by targeting SMG1 [27]. Consistent with this, our study also uncovered the high expression of miR-32-5p in OC tissues and cells. The reversal effect of miR-32-5p on circ_0078607 regulated OC progression suggested that circ_0078607 indeed sponged miR-32-5p to be involve in the regulation on OC proliferation, metastasis and apoptosis. As a target of miR-32-5p, SIK1 silencing also reversed the regulation of circ_0078607 on OC progression. In vivo experiments confirmed that circ_0078607 upregulated SIK1 to suppress OC tumorigenesis by sponging miR-32-5p.

In conclusion, our study revealed the role of circ_0078607 in OC progression and its potential new molecular mechanisms for regulating OC progression. Our data proposed that circ_0078607 restrained OC proliferation and metastasis through the miR-32-5p/SIK1 axis. These results provided new evidence for circ_0078607 as a potential therapeutic target for OC.

## Supplementary Information

**Additional file 1: Supplementary Figure 1**. The representative pictures for Fig. 6. The representative pictures for Fig. 6C (A), 6D (B), 6E (C) and 6F (D).

**Additional file 2: Supplementary Figure 2**. The representative pictures for Fig. 8. The representative pictures for Fig. 8C (A), 8D (B), 8E (C) and 8F (D).

## Data Availability

The analyzed data sets generated during the present study are available from the corresponding author on reasonable request.
