# Supplementary figures and images for: Circ_0078607 inhibits the progression of ovarian cancer via regulating the miR-32-5p/SIK1 network
Source: J Ovarian Res. 2022 Jan 4;15:3. doi: 10.1186/s13048-021-00931-9 (PMC8729016; doi:10.1186/s13048-021-00931-9)

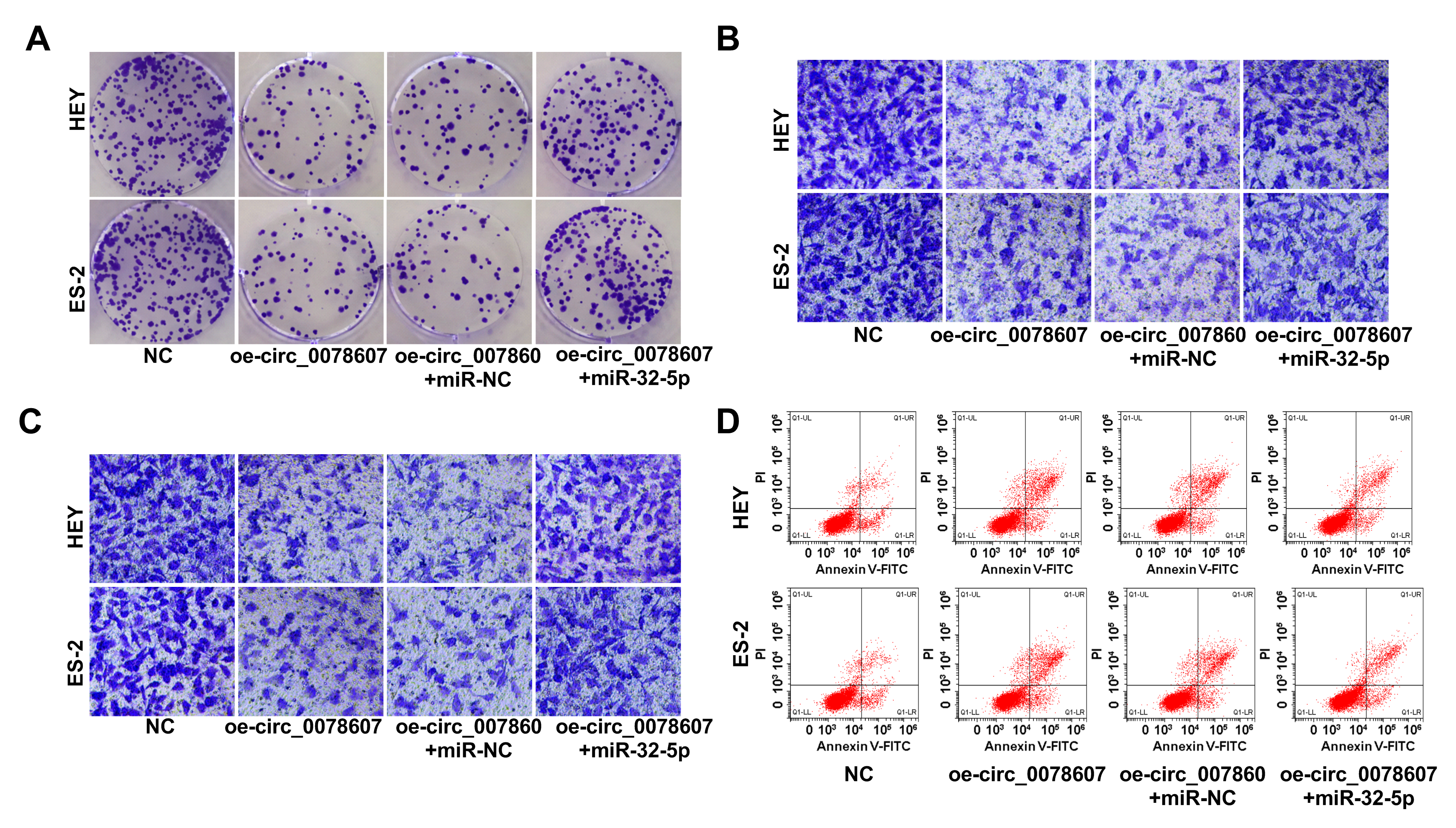

Supplement: Supplementary file 1 — Additional file 1: Supplementary Figure 1. The representative pictures for Fig. 6. The representative pictures for Fig. 6C (A), 6D (B), 6E (C) and 6F (D). [file 13048_2021_931_MOESM1_ESM.tif]

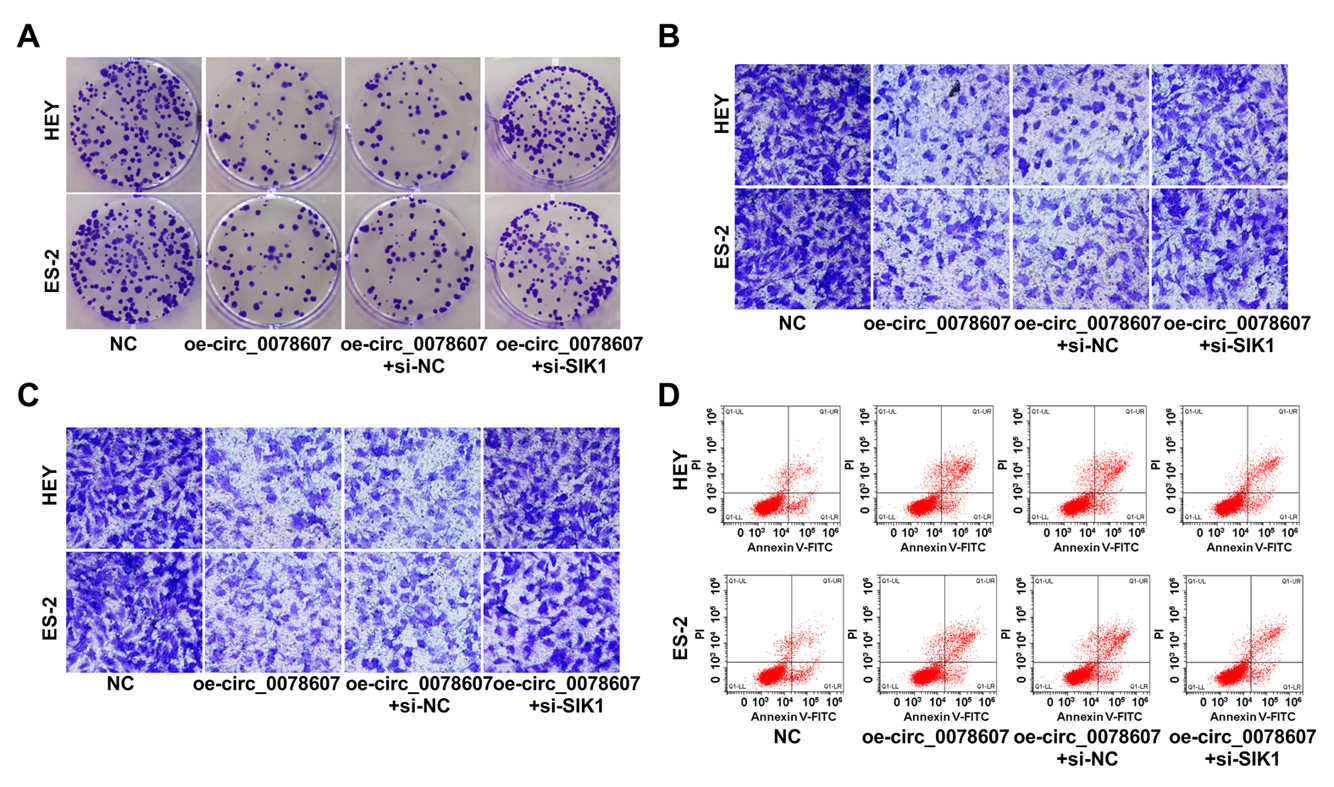

Supplement: Supplementary file 2 — Additional file 2: Supplementary Figure 2. The representative pictures for Fig. 8. The representative pictures for Fig. 8C (A), 8D (B), 8E (C) and 8F (D). [file 13048_2021_931_MOESM2_ESM.tif]
